# Establishing a three-miRNA signature as a prognostic model for colorectal cancer through bioinformatics analysis

**DOI:** 10.18632/aging.203400

**Published:** 2021-08-13

**Authors:** Yiming Wang, Lumi Huang, Nan Shan, Huiwen Ma, Songmei Lu, Xingyue Chen, Hao Long

**Affiliations:** 1 Department of Medical Oncology, Chongqing University Cancer Hospital, Chongqing, China; 2 Department of Gynaecology and Obstetrics, The First Affiliated Hospital of Chongqing Medical University, Chongqing, China; 3 Department of Palliative Care, Chongqing University Cancer Hospital, Chongqing, China

**Keywords:** CCa, miRNA, risk score, prognostic model

## Abstract

Background: Identification of more promising microRNAs (miRNAs) are being extensively studied with respect to colorectal cancer (CRC), since CRC is the leading cause of cancer deaths and most common malignant tumors worldwide. A series of colon cancer (CCa) samples from The Cancer Genome Atlas (TCGA) were analyzed to provide a new perspective into this field.

Methods: The expression of miRNAs, mRNAs and the clinical data of 437 CRC patients were downloaded from the TCGA database. The survival-related differentially expressed miRNAs (sDMIRs) and mRNAs were detected by COX regression analysis. The high-risk group and low-risk group were separated by the median risk score of the risk score model. The potential clinical characteristics of these sDMIRs were analyzed by R software. The potential molecular mechanisms of these sDMIRs were explored by computational biology. The expression levels of three sDMIRs were explored by qPCR in CRC samples.

Results: Three DMIRs (hsa-miR-21-3p, hsa-miR-194-3p and hsa-miR-891a-5p) correlated with the most remarkable prognostic values of CRC patients were selected to establish the risk score model (RSM) by univariate and multivariate COX regression analysis and the survival probability of the low-risk group was longer than that in the high-risk group. We detected the target genes of three sDMIRs and the potential molecular mechanisms of these sDMIRs. We further verified the high expression levels of hsa-miR-21-3p and hsa-miR-194-3p were associated with the early T-stages, while hsa-miR-891a-5p illustrated the reversed result.

Conclusion: Our study demonstrated three sDMIRs with significantly clinical values illustrated the potential predicting values in the prognosis of CRC patients. Our results may provide a new perspective for the diagnostic methods and treatment strategies in CRC patients.

## INTRODUCTION

Colorectal cancer (CRC) is one of the most common malignant tumors and causes the second most cancer deaths worldwide [1, 2]. At present, surgery combined with adjuvant therapies such as chemotherapy and radiotherapy is the primary strategy. However, the 5-year postoperative survival rate of CRC patients is less than 30% [3, 4]. Therefore, increasing numbers of studies try to explore promising predicting models which can help to identify CRC patients with considerable sensitivity earlier.

With the more insightful recognition of the importance of microRNAs (miRNAs), a class of approximate 20 nucleotide-long, double-stranded, non-coding RNAs (ncRNA), increasing numbers of studies have demonstrated miRNAs not only take part in post-transcriptional modifications, but also play critical regulating roles on anticancer immune responses and predicting prognosis [5, 6]. MiRNAs are potential for amending tumor cells behaviors due to abnormal gene profiles for mature and precursor sequences compared to normal tissues [7, 8]. Consisting of different tumor stromal cells, abundant ncRNA and other cytokines, tumor microenvironment (TME) is the pivotal concern of identifying prognosis-related markers [9, 10]. In the past dozen years, miRNAs have been demonstrated to be closely associated with the occurrence, development and prognosis of different tumors [11, 12]. For example, miR-21-3p has been demonstrated to be high expressed in gastric cancer, pheochromocytoma, esophageal cancer and other malignant tumors [13–15]. The attenuation of miR-17 also regulates cell death and mediates expression of SRC-3 and CLU in CRC cells [16]. MiR-194-3p highly expressed in various malignant tumors such as nasopharyngeal carcinoma, endometrial cancer and pancreatic cancer [17–19]. Besides, miR-891a-5p was found low expressed in breast cancer [20]. Therefore, we found miRNAs as a type of biomarkers in the TME, exhibit potential for predicting therapeutic sensitivity and prognosis. However, the roles of miR-21-3p, miR-194-3p and miR-891a-5p in CRC remain unclear, and few reports illuminated the clinical significance of miR-21-3p, miR-194-3p and miR-891a-5p.

The present study attempts to the clinical significance of miRNAs, especially in assessing the prognoses of CRC patients. The findings will help to open up new avenues for discovering the underlying mechanisms of miRNAs in CRC, and the RSM will also provide a novel perspective for clinical strategy making.

## MATERIALS AND METHODS

### Clinical colon samples

168 colon tumor tissues and adjacent normal tissues were obtained from the patients who performed operation in Chongqing University Cancer Hospital from September 2018 to March 2020. The obtained samples were frozen in liquid nitrogen immediately and then stored at -80° C until miRNAs extraction.

### Ethics approval and consent to participate

All patients signed the informed consent forms. The research protocol abides by the ethical principles of medical research and is approved by the Ethics Committee of Chongqing University Cancer Hospital.

### Data acquisition and differentially expressed analysis

We downed the transcriptome mRNAs and miRNAs data of CRC from The Cancer Genome Atlas (TCGA) data portal (https://portal.gdc.cancer.gov/). The data in the present study were currently updated on May 7, 2020. We excluded the data of patients with the overall survival (OS) of CRC patients ≤30 days, because these patients probably died from other causes). We then acquired the genes with the differential expression in tumor tissues and adjacent normal tissues by the limma package of R software (https://bioconductor.org/packages/release/bioc/html/limma.html). The screening value of all differentially expressed mRNAs and miRNAs is “FDR < 0.05, log_2_| FC | >1 and P < 0.05”.

### Survival-related differentially expressed miRNAs (sDMIRs) and the risk score model (RSM)

Differentially expressed miRNAs (DMIRs) with significant clinical outcomes were served as survival-related DMIRs (sDMIRs). We utilized univariate COX regression analysis to screen sDMIRs (*P* < 0.001) and the value of hazard ratio confirmed sDMIRs into protective and deleterious parts. The sDMIRs were further analyzed by the multivariate COX regression analysis, and were employed to establish the RSM. RSM was built by the expression levels multiplied by Cox regression coefficients. Based on the median risk score of RSM, CRC patients were separated into the high-risk group and the low-risk group. We further analyzed the relevance of the RSM and clinical features by T-test to detect the clinical application value of the sDMIRs.

### Real-time quantitative PCR

The PrimeScript RT reagent kit (TaKaRa, Osaka, Japan) was used to reverse-transcribe RNAs to cDNAs. The reaction steps [21] were shown as follows: 37° C for 15 min and 85° C for 5s. The quantitative polymerase chain reaction (qPCR) was run on an ABI 7500 Real-Time PCR System (Applied Biosystems) using a SYBR Green assay (TaKaRa). The reaction cycling conditions were 95° C for 30 s, 40 cycles of |95° C for 5 s, and 60° C for 34 s; primer sequences are shown in Table 1. The relative quantification levels of miRNAs were standardized to U6 by the 2^−ΔCt^ method. Each cDNA sample was replicated three assays.

**Table 1:** The primer sequences of hsa-miR-21-3p, hsa-miR-194-3p, and hsa-miR-891a-5p.

**hsa-miR-21-3p**	F primer(5’-3’)	GACCCAACACCAGTCGATG
	R primer(5’-3’)	TCCTCCTCTCCTTCCTTCTC
	RT(5’-3’)	GTCCTCCTCTCCTTCCTTCTCATGAGGAGGACACAGCC
**hsa-miR-194-3p**	F primer(5’-3’)	CCAGTGGGGCTGCTGT
	R primer(5’-3’)	GAGAGGAGAGGAAGAGGGAA
	RT(5’-3’)	GGAGAGGAGAGGAAGAGGGAAATCTCCTCTCCCAGATA
**hsa-miR-891a-5p**	F primer(5’-3’)	GTGCAACGAACCTGAGC
	R primer(5’-3’)	GACCTGAACCTGAACCTGAA
	RT(5’-3’)	GGACCTGAACCTGAACCTGAAATTTCAGGTCCTCAGTG
**U6**	F primer(5’-3’)	CTCGCTTCGGCAGCACA
	R primer(5’-3’)	AACGCTTCACGAATTTGCGT
	RT(5’-3’)	AAAATATGGAACGCTTCACGAATTTG

Note: F primer, forward primer; R primer, reverse primer; RT, reverse transcription.

### Bioinformatics analysis

We employed the miRTarBase (https://mirtarbase.cuhk.edu.cn/~miRTarBase/miRTarBase_2019/php/index.php), TargetScan (http://www.targetscan.org/vert_72/) and miRDB (http://mirdb.org/) databases to screen the target genes (TGs). A target gene needs to meet a standard of two databases at least. In order to further investigate these TGs, the GO and KEGG pathways were employed to discover the underlying mechanisms. The receiver operating characteristic (ROC) curve was generated by the R software. Univariate and multivariate Cox regression analysis were employed to confirm the sDMIRs. The survival probability of CRC patients was assessed by Kaplan-Meier survival curve.

### Statistical analysis

GraphPad Prism5 (GraphPad Software Inc, La Jolla, CA, USA) and SPSS19.0 software (SPSS Inc, Chicago, IL, USA) were utilized for statistical analyses. The clinical correlations were determined by ANOVA, post-hoc test (Boferroni method) and independent T-test. *P* < 0.05 was considered statistically significant.

### Availability of data and materials

Authors can provide all of datasets analyzed during the study on reasonable request.

## RESULTS

### MiRNAs and mRNAs with differential expression

**Figure 1:**
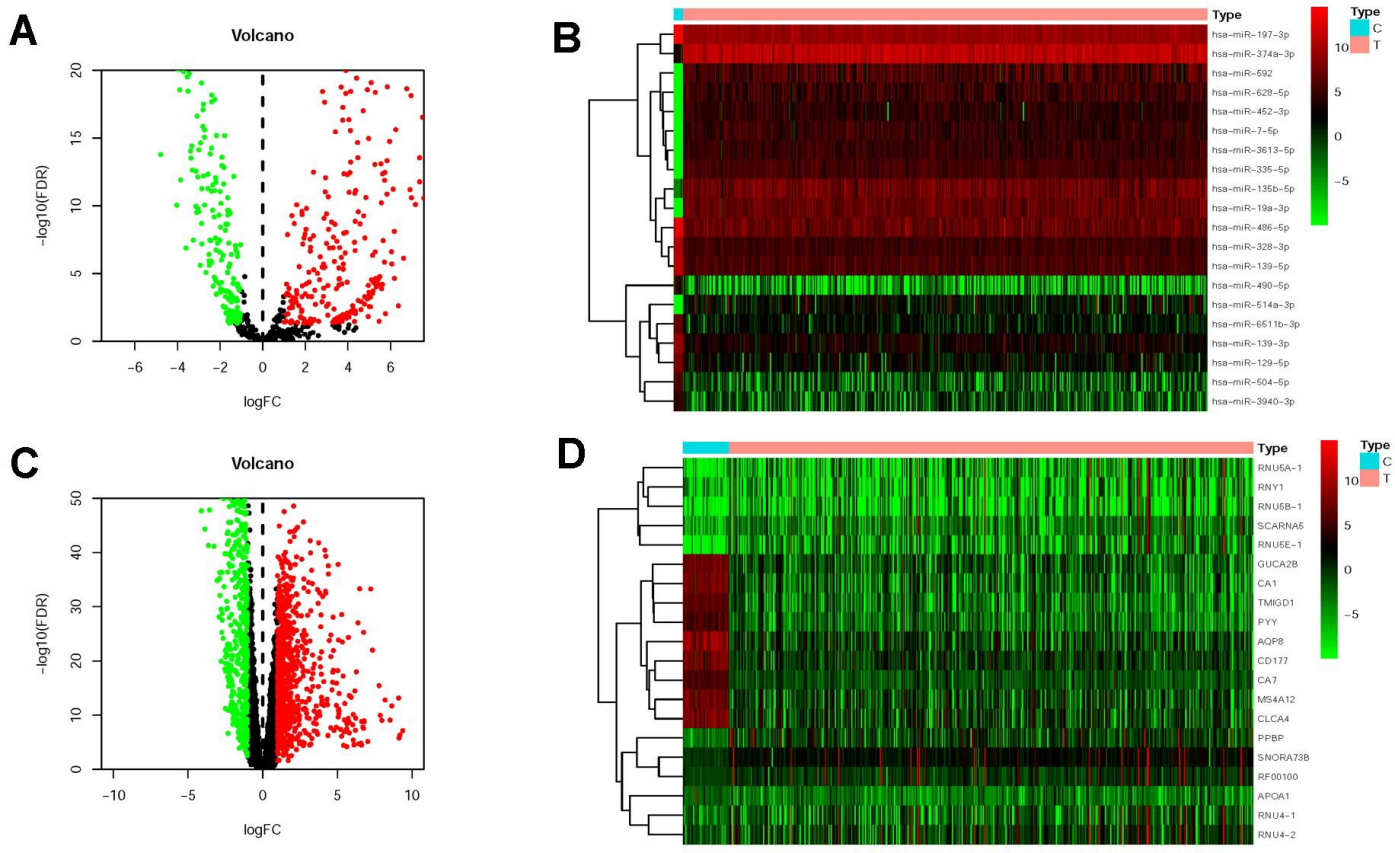
**Differentially expressed miRNAs and mRNAs in CRC.** The differentially expressed miRNAs (DMIRs) in CRC and adjacent non-tumor tissues were showed in the volcano plot (**A**) and heatmap (**B**) based on TCGA database. Volcano plot (**C**) and heatmap (**D**) demonstrated the differentially expressed mRNAs between CRC tissues and adjacent normal tissues based on TCGA database. The red parts represented the upregulated genes; the green parts represented the downregulated genes, and the black parts represented the genes without significant difference. FDR < 0.05, log_2_ | FC | >1 and *P* < 0.05.

Through limma algorithm, we identified 496 differentially expressed miRNAs (DMIRs), of which 212 were downward and 285 were raised (Figure 1A and Supplementary Table 1). And the 10 most downward and raised miRNAs were illustrated in the heatmap by the value of log_2_|FC| (Figure 1B). Next, 1533 CRC genes with differential expression (DMRs) were also identified, including 651 downward and 882 raised mRNAs (Figure 1C). The 10 most downward and raised mRNAs were shown in Figure 1D.

### The relevancies between DMIRs and clinical prognosis

**Figure 2:**
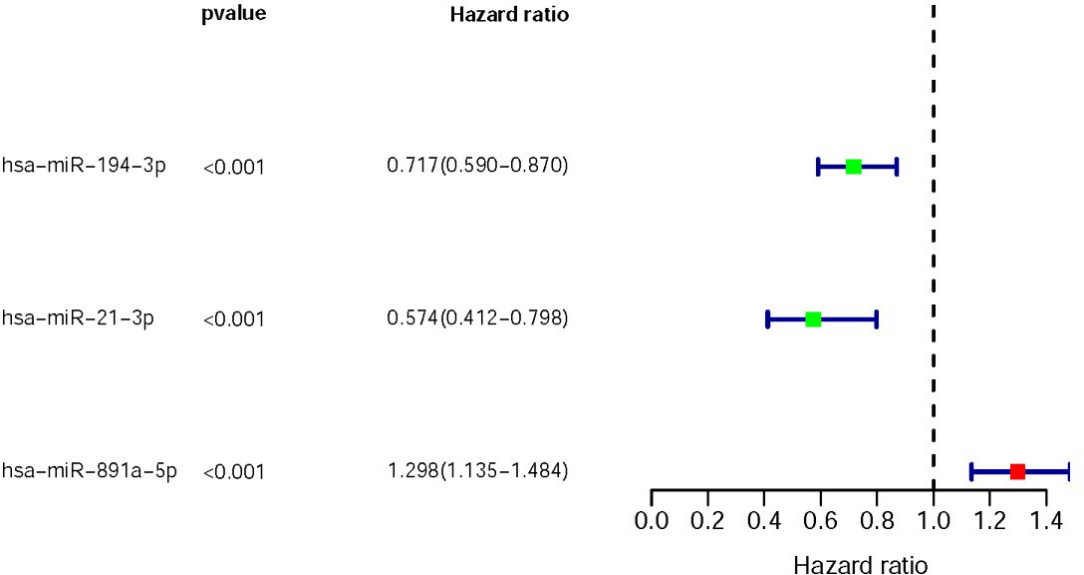
**DMIRs with significant clinical outcomes.** The forest plot showed the survival-related hazard ratio of sDMIRs (hsa-miR-194-3p, hsa-miR-21-3p and hsa-miR-891a-5p) based on TCGA database. Red parts represented the up-regulation, and green parts represented the down-regulation.

We selected 3 DMIRs which were associated with the clinical prognoses of CRC patients (sDMIRs), including hsa-miR-194-3p, hsa-miR-21-3p and hsa-miR-891a-5p. The relationships between these sDMIRs and clinical prognosis were shown in Figure 2, of which has-miR-891a-5p showed a positive relation with the poor clinical prognosis, but hsa-miR-194-3p and hsa-miR-21-3p were illustrated negative correlations with the poor clinical prognosis. Three sDMIRs (hsa-miR-891a-5p, hsa-miR-21-3p and hsa-miR-194-3p) were identified by the multivariate COX regression analysis to establish the risk score model (RSM). The formula was as followed, [Expression levels of hsa-miR-891a-5p* (0.190971)] + [Expression of hsa-miR-194-3p * (-0.220654)] + [Expression of hsa-miR-21-3p * (-0.453552)]. As shown in the survival curve, the high level of hsa-miR-891a-5p indicated the poor prognosis, while the hsa-miR-21-3p and hsa-miR-194-3p showed positive relations with OS (Supplementary Figure 1).

### Clinical features and application of RSM

**Figure 3:**
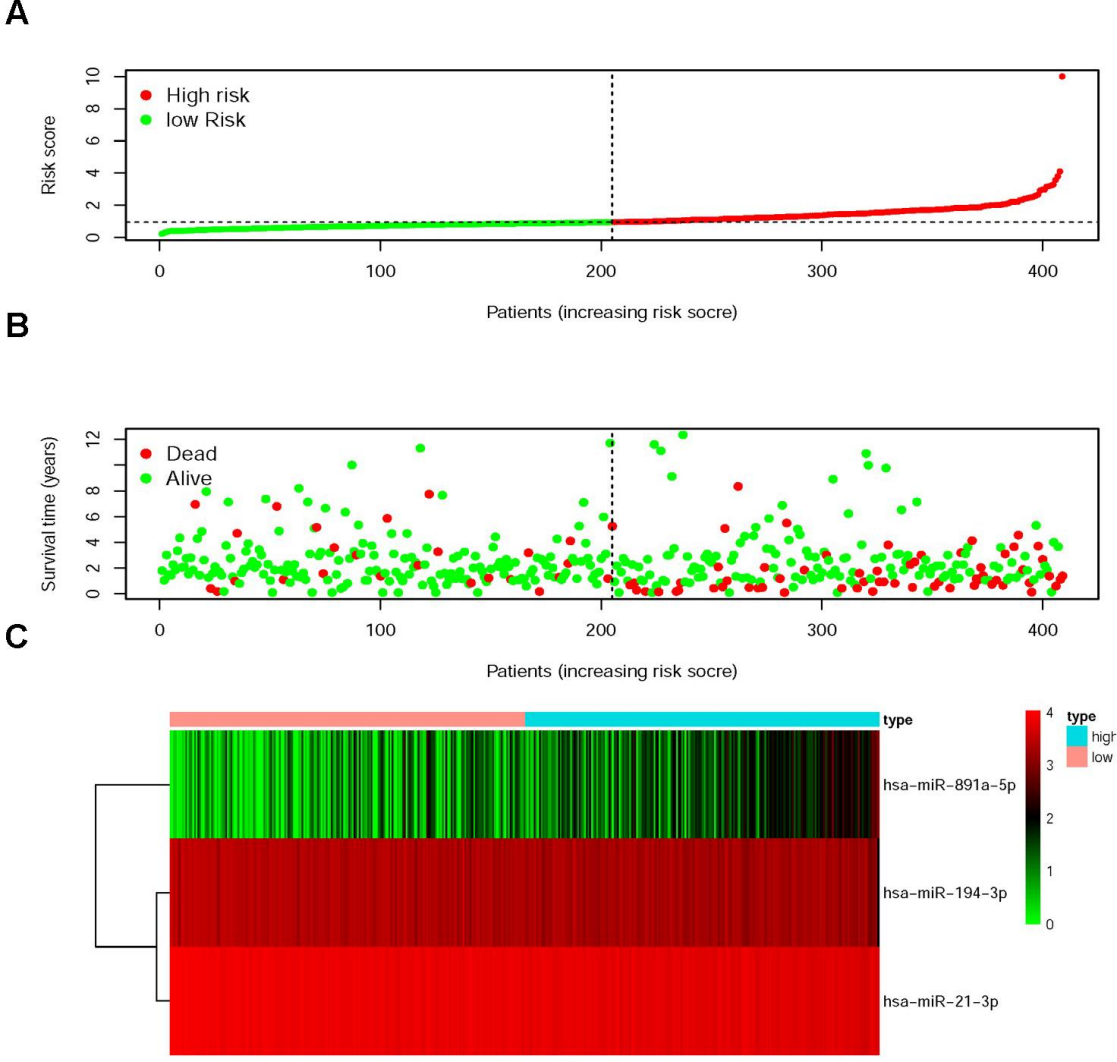
**Risk score model (RSM) was established based on sDMIRs.** The distribution of risk score in the high-risk group and the low-risk group (**A**). Survival status in the low-risk group and the high-risk group (**B**). The heatmap of the expression levels of sDMIRs (hsa-miR-194-3p, hsa-miR-21-3p and hsa-miR-891a-5p) contained in the RSM (**C**) based on TCGA database.

**Figure 4:**
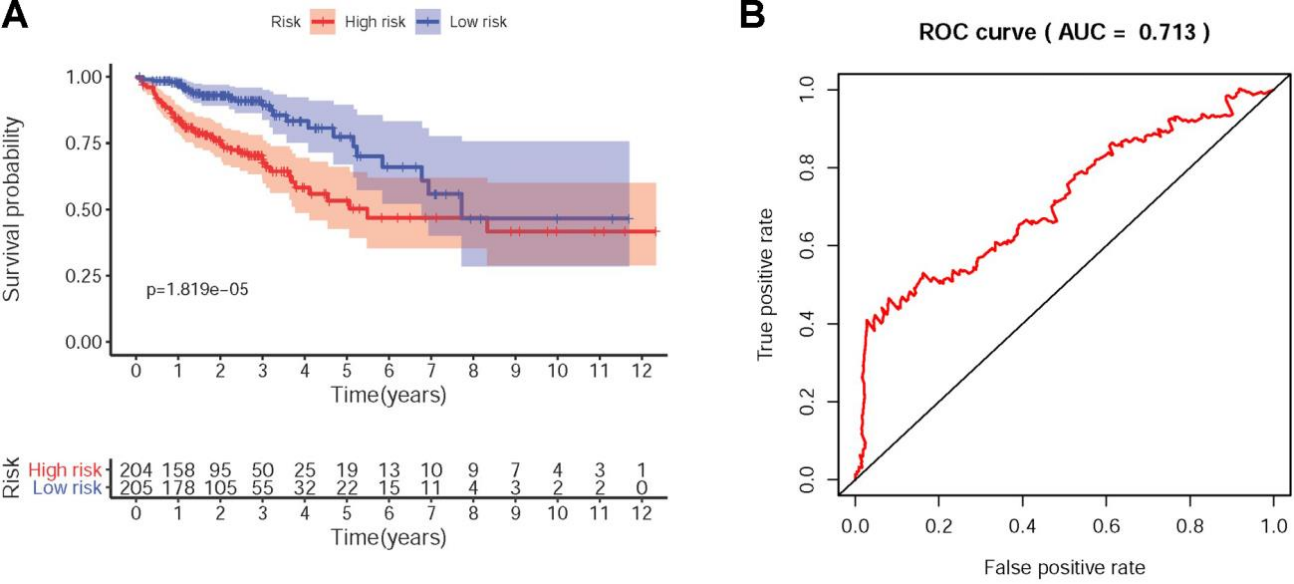
**The survival curve and receiver operating characteristic (ROC) curve of RSM.** Kaplan-Meier survival curve of OS in the high-risk group and the low-risk group (**A**). The high-risk group showed the poor prognoses in the CRC patients based on TCGA database. The ROC curve relevance the accuracy of the RSM (**B**) and the area under curve (AUC) was 0.713 based on TCGA database.

**Figure 5:**
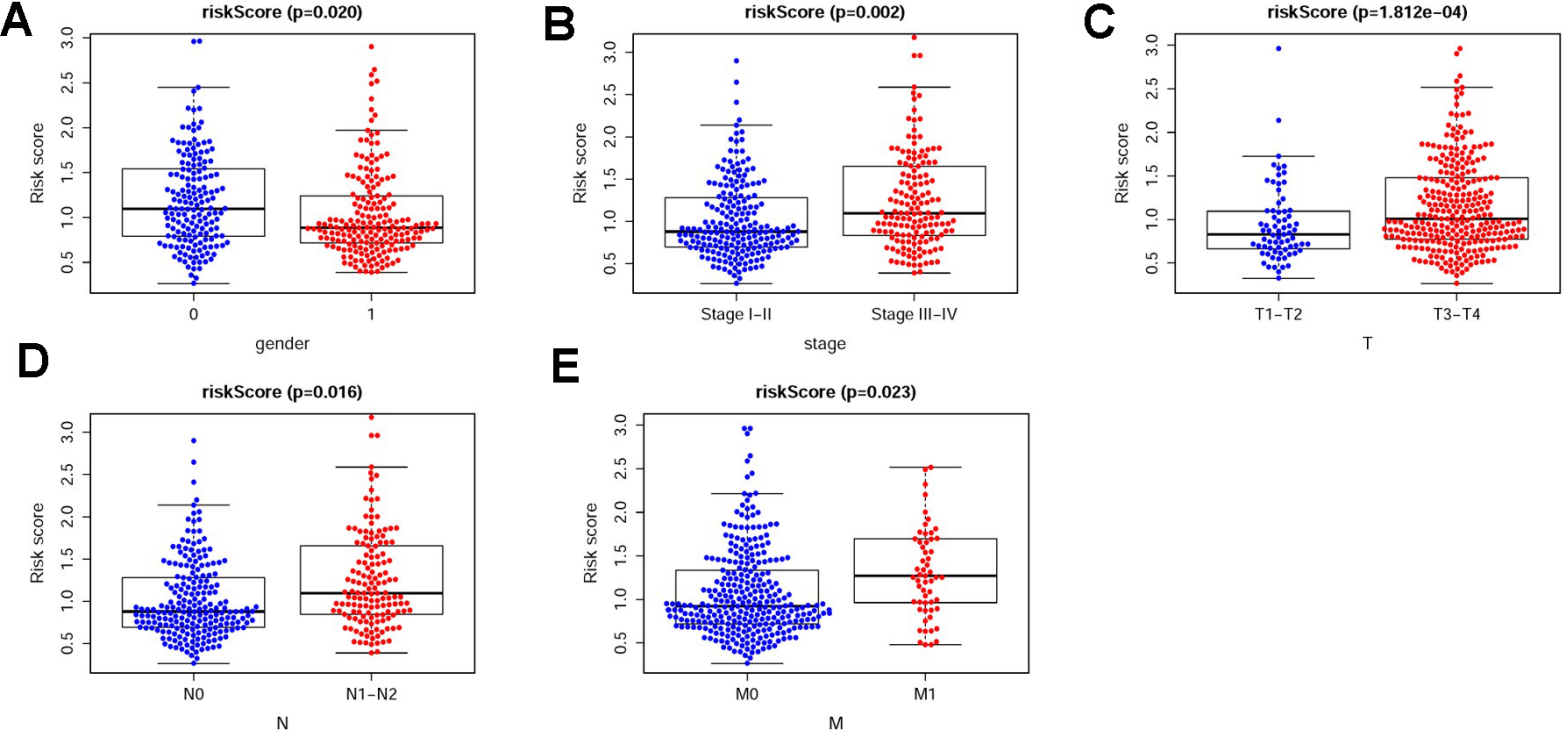
**The relevancies between the clinical features and the RSM.** The relevancies between the RSM and gender (**A**), stage (**B**), T-stage (**C**), N-stage (**D**) and M-stage (**E**). The lower risk scores were showed in the male patients (**A**), and patients with early stage (**B**), early T-stage (**C**), early N-stage (**D**) and early M-stage (**E**) based on TCGA database. (0 = Female patients; 1 = Male patients).

**Figure 6:**
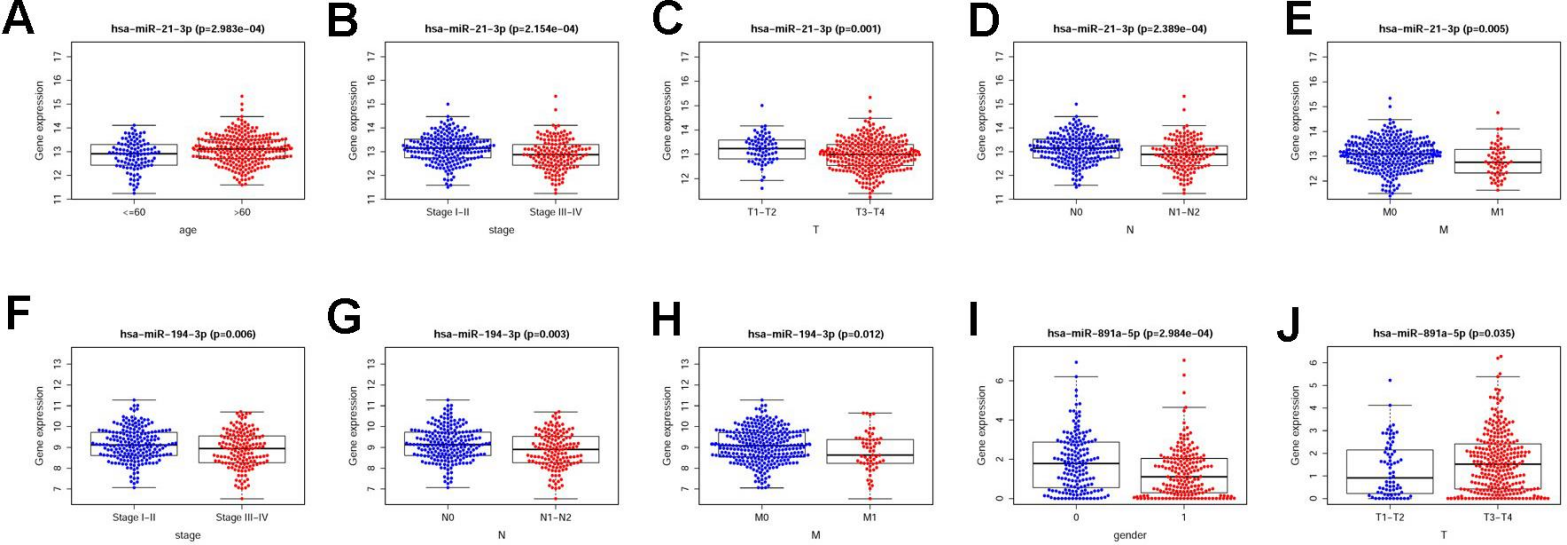
**The relevancies between the clinical features and sDMIRs.** The expression levels of hsa-miR-21-3p were increased in older patients (**A**), patients with early stage (**B**), early T-stage (**C**), early N-stage (**D**) and early M-stage (**E**). The expression levels of hsa-miR-194-3p were decreased in the patients with advanced stage (**F**), advanced N-stage (**G**) and advanced M-stage (**H**). The female patients (**I**) and patients with advanced T-stage (**J**) were correlated with the higher expression levels of hsa-miR-891a-5p based on TCGA database. (0 = Female patients; 1 = Male patients).

The sDMIRs were used to establish RSM, and the CRC patients were divided into the low-risk group and the high-risk group (Figure 3A). As illuminated in the Figure 3B, the mortality decreased with the lower risk score. Besides, with the increased risk score, the expression of hsa-miR-891a-5p rather than hsa-miR-21-3p and hsa-miR-194-3p was prominently enhanced (Figure 3C). The high-risk group showed the poorer OS than the low-risk group (Figure 4A). The ROC curve was employed to investigate the accuracy of RSM, and the area under curve of the ROC curve was 0.713, meaning the RSM in survival prediction was satisfied (Figure 4B). To further investigate the clinical application of RSM and the sDMIRs, we detected the relevance between the RSM and the clinical features, such as age, gender, and TNM-stage. We found that female (Figure 5A), advanced stage (Figure 5B) and advanced TNM-stage (Figure 5C–5E) were the remarkable risk factors with the higher scores. Next, we further analyzed the relationships between sDMIRs and the clinical features. We found that the early young (Figure 6A), advanced stage (Figure 6B), and advanced TNM-stage (Figure 6C–6E) were correlated with the lower expression level of hsa-miR-21-3p. Besides, hsa-miR-194-3p expressed decreasingly with the advanced stage (Figure 6F) and advanced NM-stage (Figure 6G, 6H). The male patients (Figure 6I) and patients with early T-stage (Figure 6J) showed the negative correlations with the increased expression level of hsa-miR-891a-5p.

### The TGs of the sDMIRs and functional enrichment analysis

**Figure 7:**
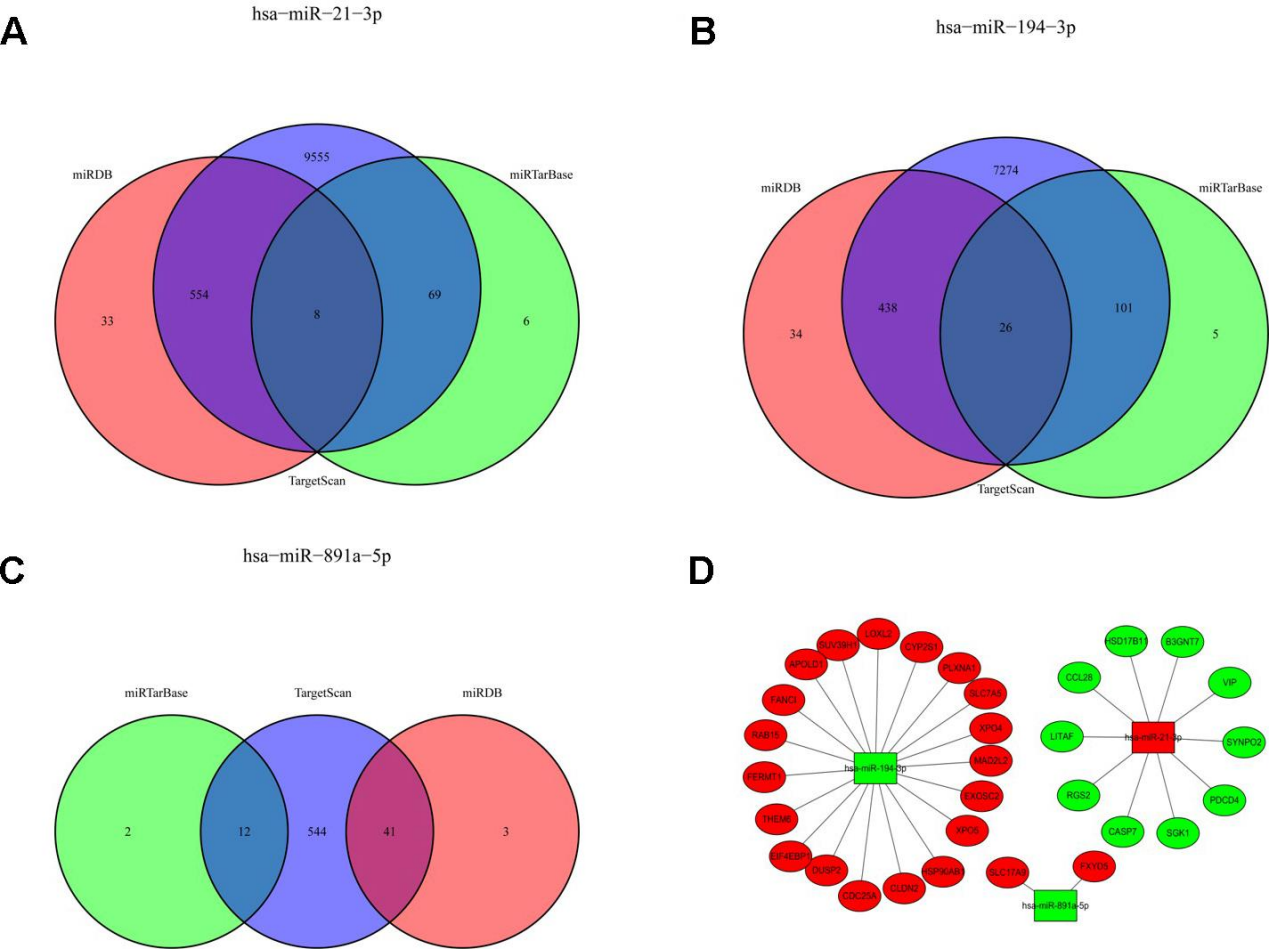
**The Venn diagram of the sDMIRs TGs and the regulatory networks of sDMIRs.** The predicted TGs were screened from miRDB, TargetScan, and miRTarBase databases. The overlaps meant the numbers of TGs predicted by more than one database. ((**A**) hsa-miR-21-3p; (**B**) hsa-miR-194-3p; (**C**) hsa-miR-891a-5p). The regulatory networks of sDMIRs and TGs (**D**); green parts represent down-regulation and red parts represent up-regulation.

**Figure 8:**
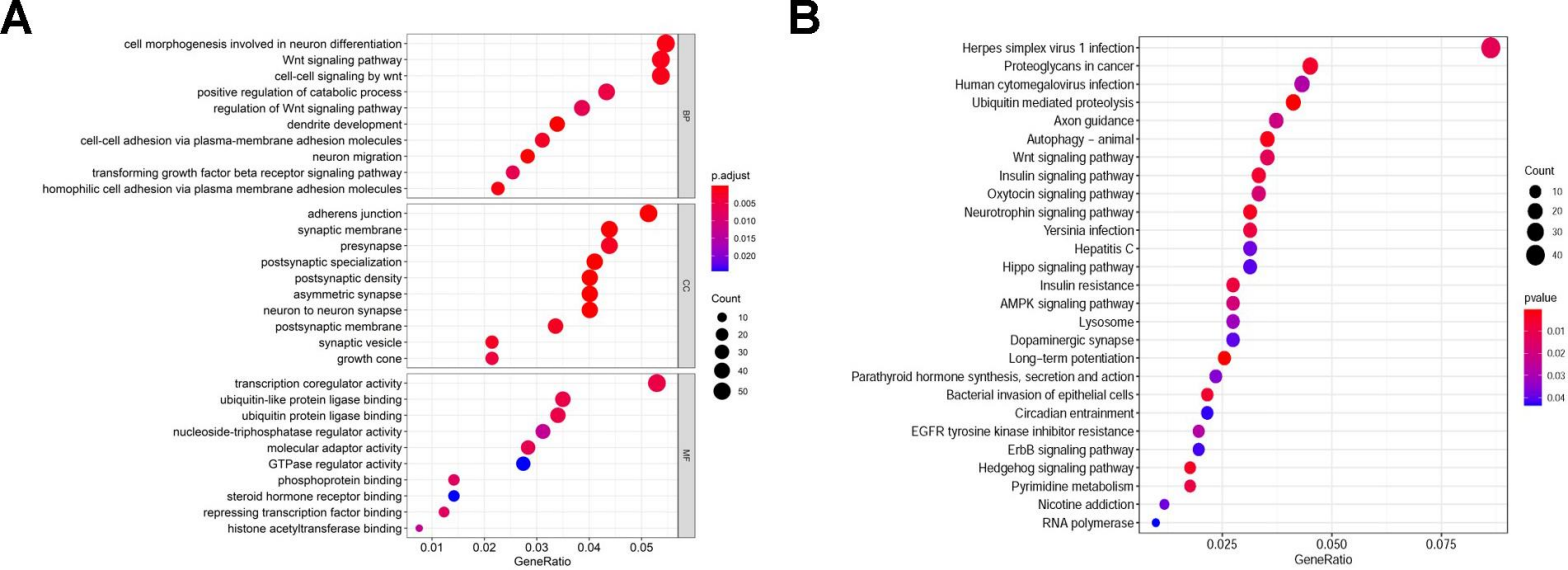
**The functional enrichment analysis of TGs.** The top pathways of TGs were demonstrated in biological process (BP), cellular component (CC), molecular function (MF) (**A**), and KEGG pathway (**B**).

In order to detect the TGs of sDMIRs, we predicted and screened the TGs by the databases of TargetScan, miRTarBase and miRDB (Figure 7A–7C). Meanwhile, Figure 7D illustrated the regulatory net among the sDMIRs and TGs. Additionally, we utilized the GO and KEGG analyses to investigate the related molecular mechanisms of these TGs. We found that, in the biological processes, cellular components and molecular function, “cell morphogenesis involved in neuron differentiation”, “adherens junction” and “transcription coregulator activity” were the most significant enrichments, respectively (Figure 8A). In KEGG analysis, “Herpes simplex virus 1 infection” was the most enriched (Figure 8B).

### MiR-21-3p, miR-194-3p and miR-891a-5p obtained the prominent clinical significance

**Figure 9:**
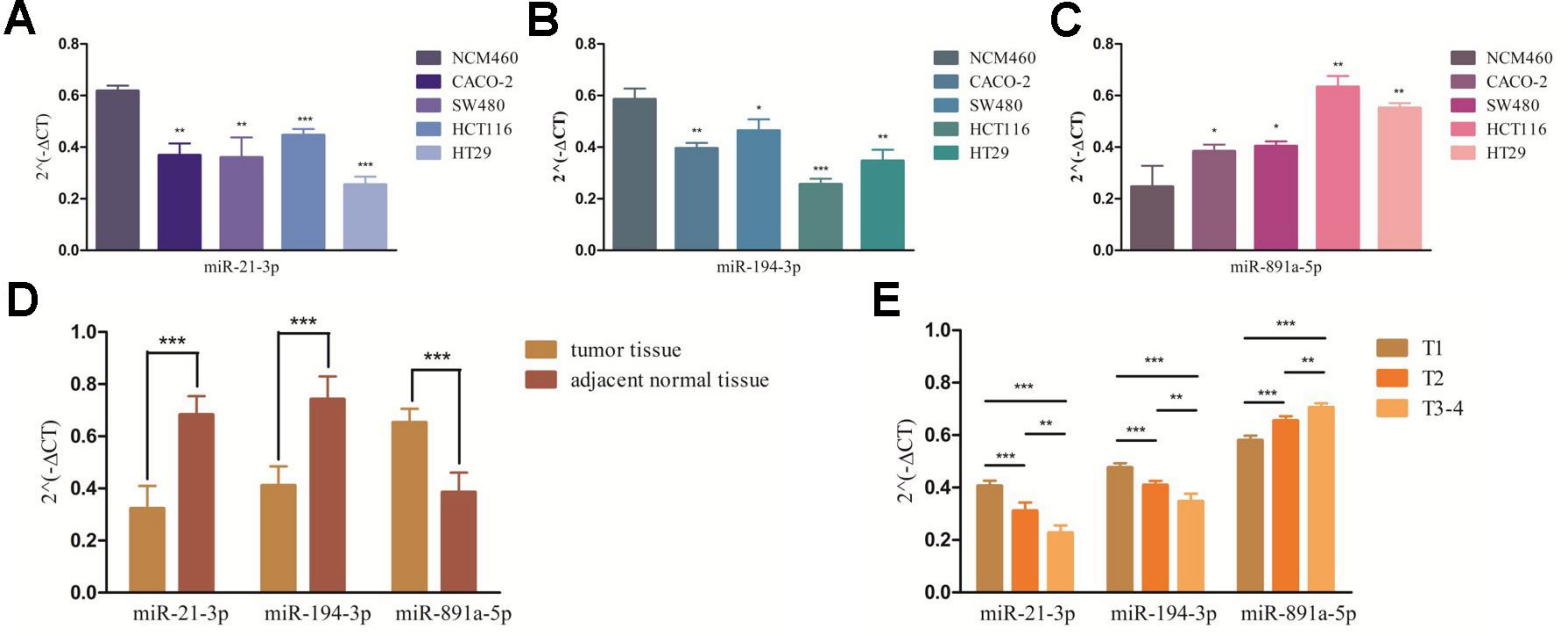
**The expression levels of miR-21-3p, miR-194-3p and miR-891a-5p in patients with CCa and its correlation with T-stages.** The results of RT-qPCR of miR-21-3p’ (**A**), miR-194-3p’ (**B**) and miR-891a-5p’ (**C**) expression levels in colonic cancer cell lines. ***, ** and * represent the remarkable difference compared with NCM460 (*P*<0.001, *P*<0.01 and *P*<0.05), respectively. The expression levels of miR-21-3p, miR-194-3p and miR-891a-5p in carcinoma tissues and adjacent tissues based on the CRC samples of Chongqing University Cancer Hospital Clinical (**D**). *** Represent the significant difference compared with adjacent tissues (*P*<0.001). The expression levels of miR-21-3p, miR-194-3p and miR-891a-5p in CCa tissues with various T-stages based on the CRC samples of Chongqing University Cancer Hospital Clinical (**E**). ***, ** and * represent the significant difference between groups (*P*<0.001, *P*<0.01 and *P*<0.05), respectively.

To further verify the clinical significance of the RSM, we tested the expression and clinical relevance of miR-21-3p, miR-194-3p and miR-891a-5p which were involved in the RSM *in vivo* and *in vitro*. As showed in Figure 9, miR-21-3p (Figure 9A) and miR-194-3p (Figure 9B) expressed lowly in CRC cell lines than that in the colonic epithelial cell, but miR-891a-5p (Figure 9C) showed the reversed results. Additionally, we investigated the relevance of the three sDMIRs with T-stage. We found that, compared to adjacent tissues, the expression of miR-891a-5p rather than miR-21-3p and miR-194-3p was higher in carcinoma tissue (Figure 9D). Additionally, the gradually higher expression of miR-891a-5p was tested in tumor tissues of CRC patients with more advanced T-stage, and miR-21-3p and miR-194-3p expressed decreasingly as the more advanced T-stages (Figure 9E).

## DISCUSSION

Recently, accumulating miRNAs were validated to play pivotal roles on diagnosis and prediction in the field of cancer. Gmerek detected a cascade of 40 miRNAs with differential expression in cancerous, of which 8 miRNAs could be used as potential biomarkers for diagnosis of CRC patients [22]. Tang found miRNA-320d in tumor-derived exosome could be utilized as a marker for metastatic CRC [23]. Given the values of miRNAs on diagnosis and prognosis prediction, discovering more promising and sensitive biomarkers such as DMIRs attracted increasing attentions.

In the past few years, increasing studies illuminated the crucial roles of TME in the prognoses of patients. Hinshaw found the TME innately modulates cancer progression [24]. Therefore, more researchers began to turn their eyes on the roles of TME. Besides, large quantities of studies have identified various DMIRs with the significant assessing potential for CRC [3, 25, 26]. Although amounts of studies of various cancers have elucidated the clinical significance of DMIRs, especially in tumor invasion, metastasis and prognosis, the complete genome-wide analysis of CRC remain insufficiency, especially on the mechanisms [27–29]. Sun highlighted that miR-21-3p could promote the progression of gastric cancer [13]. Bruna Calsina revealed that miR-21-3p, as a biomarker, was potential for risk stratification to improve the management of patients with pheochromocytomas and paragangliomas [14]. Besides, miR-194-3p has been demonstrated to stimulate the occurrence and progression of breast cancer [17]. However, the roles of miR-21-3p, miR-194-3p and miR-891a-5p in predicting prognosis of CRC remain unclear. It has been reported that miR-21-3p promoted cell proliferation, migration and invasion through regulating TSC2/mTOR pathway, and augmented tumor proliferation via PI3K/AKT pathway [14, 30]. MiR-194-3p strengthened cell proliferation, and migration through targeting and regulating the protein regulator of cytokinesis-1 and methyl CpG binding protein-2 [17, 19]. MiR-891a-5p could inhibited tumor cell proliferation, migration and invasion by NFkB pathway [31].

In the present study, we analyzed the expression of mRNAs and miRNAs in 437 CRC patients in TCGA. Next, we identified 3 sDMIRs used to establish the RSM, in which the high-risk group had the poorer OS. The female and advanced stage and TNM-stage got the higher risk scores in RSM. Although female CRC patients have a lower incidence than male CRC patients, the morbidity and mortality ratio of female patients is slightly higher than that of male patients. Hence, this may be the reason why women have higher risk score than men [32]. We established the regulation networks of TGs and three sDMIRs to find the TGs of three sDMIRs. Besides, we analyzed the TGs by the KEGG and GO analysis to detect the potential molecular mechanisms. In KEGG analysis, “Herpes simplex virus 1 infection” was the most enriched. In the biological processes, cellular components and molecular function, “cell morphogenesis involved in neuron differentiation”, “adherens junction” and “transcription coregulator activity” were the most significant enrichments, respectively. These results of mechanisms need to be verified by more experiments in the future.

In order to enhance the reliability and persuasion of our RSM, we recruited some patients with CRC. We detected the expression of sDMIRs with obvious clinical significances in CRC clinical samples. The expression of miR-891a-5p was lower in adjacent tissues than that in carcinoma tissues, but miR-21-3p and miR-194-3p expressed highly in adjacent tissues. Besides, the higher expression of miR-891a-5p was related to more advanced T-stage, but miR-21-3p and miR-194-3p showed the reversed results.

Although we demonstrated the clinical significance of RSM in assessing prognoses of CRC patients and tested the expression of sDMIRs involved in RSM, certain limitations are still needed to be improved in future studies. First, multiple omics analysis should be conducted to better ascertain the significance and mechanisms of sDMIRs. Then, *in vivo* and *in vitro* detections of other sDIMRs involved in RSM should also be completed.

## CONCLUSIONS

In this study, we demonstrated the clinical significance of the RSM which was established base on sDMIRs, especially in assessing prognoses of CRC patients. These findings open up new avenues for clinical decisions and provide an accurate and novel model for prognostic evaluation of CRC patients.

## Supplementary Material

Supplementary Figure 1

Supplementary Table 1
