# Supplementary material for: Establishing a three-miRNA signature as a prognostic model for colorectal cancer through bioinformatics analysis
Source: Aging (Albany NY). 2021 Aug 13;13(15):19894–907. doi: 10.18632/aging.203400 (PMC8386531; doi:10.18632/aging.203400)
Supplement: Supplementary Figure 1 [file aging-13-203400-s001.pdf]

SUPPLEMENTARY FIGURE

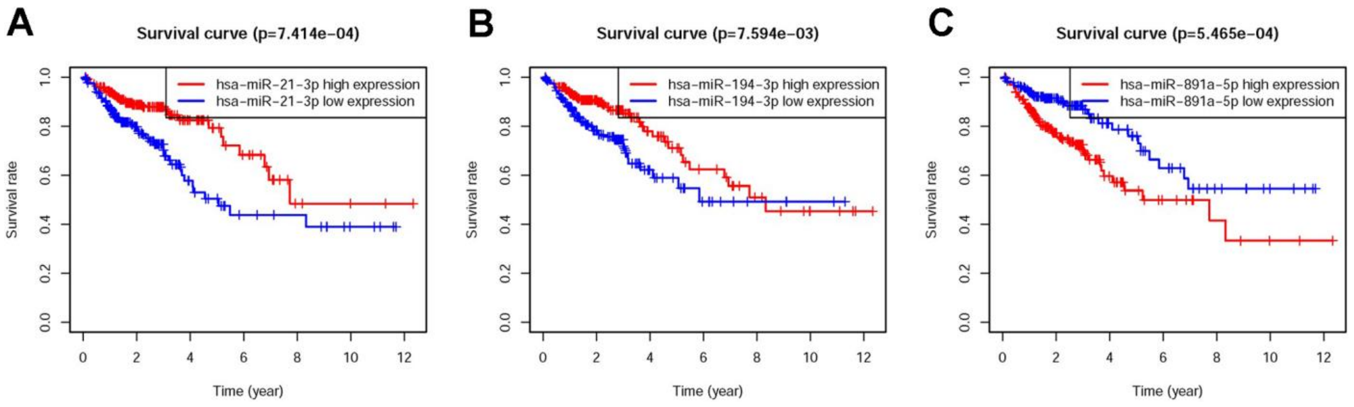

Supplementary Figure 1. Survival curve of sDMIRs. Kaplan-Meier survival curve of sDMIRs in CRC patients. The higher expression levels of hsa-miR-21-3p (**A**) and hsa-miR-194-3p (**B**) were correlated with the longer OS based on TCGA database. The higher expression levels of hsa-miR-891a-5p (**C**) were related with the poor prognosis based on TCGA database.
